# *CFTR* Mutations Spectrum and the Efficiency of Molecular Diagnostics in Polish Cystic Fibrosis Patients

**DOI:** 10.1371/journal.pone.0089094

**Published:** 2014-02-26

**Authors:** Ewa Ziętkiewicz, Ewa Rutkiewicz, Andrzej Pogorzelski, Barbara Klimek, Katarzyna Voelkel, Michał Witt

**Affiliations:** 1 Department of Molecular and Clinical Genetics, Institute of Human Genetics, Polish Academy of Sciences, Poznan, Poland; 2 Department of Pneumology and Cystic Fibrosis, Institute of Tuberculosis and Lung Diseases, Rabka, Poland; 3 International Institute of Molecular and Cell Biology, Warsaw, Poland; Odense University Hospital, Denmark

## Abstract

Cystic fibrosis (CF) is caused by mutations in the cystic fibrosis transmembrane regulator gene (*CFTR*). In light of the strong allelic heterogeneity and regional specificity of the mutation spectrum, the strategy of molecular diagnostics and counseling in CF requires genetic tests to reflect the frequency profile characteristic for a given population. The goal of the study was to provide an updated comprehensive estimation of the distribution of *CFTR* mutations in Polish CF patients and to assess the effectiveness of INNOLiPA_*CFTR* tests in Polish population. The analyzed cohort consisted of 738 patients with the clinically confirmed CF diagnosis, prescreened for molecular defects using INNOLiPA*_CFTR* panels from Innogenetics. A combined efficiency of INNOLiPA *CFTR_19* and *CFTR_17_TnUpdate* tests was 75.5%; both mutations were detected in 68.2%, and one mutation in 14.8% of the affected individuals. The group composed of all the patients with only one or with no mutation detected (109 and 126 individuals, respectively) was analyzed further using a mutation screening approach, i.e. SSCP/HD (single strand conformational polymorphism/heteroduplex) analysis of PCR products followed by sequencing of the coding sequence. As a result, 53 more mutations were found in 97 patients. The overall efficiency of the CF allele detection was 82.5% (7.0% increase compared to INNOLiPA tests alone). The distribution of the most frequent mutations in Poland was assessed. Most of the mutations repetitively found in Polish patients had been previously described in other European populations. The most frequent mutated allele, F508del, represented 54.5% of Polish CF chromosomes. Another eight mutations had frequencies over 1%, 24 had frequencies between 1 and 0.1%; c.2052-2053insA and c.3468+2_3468+3insT were the most frequent non-INNOLiPA mutations. Mutation distribution described herein is also relevant to the Polish diaspora. Our study also demonstrates that the reported efficiency of mutation detection strongly depends on the diagnostic experience of referring health centers.

## Introduction

Cystic fibrosis (CF; MIM 219700) is the most frequent autosomal recessive disease among Caucasians; its median incidence in Europe is 1 in 3,500 [1] and ranges from 1 in 1,350 to 1 in 25,000, depending on the population under study [2]. The disease is caused by mutations in the cystic fibrosis transmembrane regulator (*CFTR*) gene (Ensembl ENSG00000001626) [3]. The comprehensive list of the *CFTR* mutations, maintained at the Cystic Fibrosis Mutation Database (CFMDB) (www.genet.sickkids.on.ca/cftr), was approximately 1950 as of November 2013.

The most frequent *CFTR* mutation, F508del, accounts for ∼66% CF chromosomes in the general Caucasian population [4]–[5]. Few other widely spread mutations reach worldwide frequencies >1%, less than twenty have frequencies between 0.1 and 1.0%, and the majority are found only in certain geographical regions, populations or “private” (reported in singular families). Local frequencies of the most common mutations vary among populations [4]–[5], with founder effect(s) shown to be responsible for a number of them. The strong allelic heterogeneity has a direct bearing on the strategy of molecular diagnostics and counseling in CF. In order to increase the rate of *CFTR* mutation detection and to correctly evaluate the residual risk of being a CF carrier after molecular analysis, it is essential that genetic tests are designed based on the frequency profile characteristic for a given population and that the sensitivity to detect mutations is as high as possible [4]–[6].

The most recent publication regarding the prevalence of *CFTR* mutations in Poland is now more than 12 years old [7]; the other three papers investigated only a limited number of mutations [8]–[9] or a specific subpopulation of adult CF patients [10].

The goal of the present study was to provide an updated comprehensive estimation of the distribution of *CFTR* mutations in Poland, based on the analysis of a representative cohort of CF patients. At the same time, we aimed to assess the effectiveness of INNOLiPA (IL) *CFTR* tests (Innogenetics) in PL (Polish) population.

## Results

Two IL tests, *CFTR_19* and *CFTR_17 TnUpdate*, used to examine a cohort of 738 PL CF patients, revealed mutations in 66.8% and 8.7% of the CF alleles, respectively (a combined efficiency of 75.5%). Two mutations were found in 68.2%, and of one mutation in 14.8% of the patients, and no mutation was identified in 17.1% of PL CF patients (Table 1). Of the 36 most frequent European-wide *CFTR* mutations targeted by both IL tests, 22 were found in at least one individual (Table 2).

**Table 1:** Mutation detection efficiency using INNOLiPA tests*: CFTR19* and *CFTR17TnUpdate*.

Mutations detected in a patient	Number of CF patients	Alleles with no mutation identified	Alleles with CF mutations detected using INNOLiPA tests		Further molecular analysis
			*CFTR*_19	*CFTR*_17*TnUpdate^a^*	
both	503 (68.1%)	0	888 (60.2%)	118 (8.0%)	Not necessary
one	109 (14.8%)	109	98 (6.6%)	11 (0.7%)	Yes
none	126 (17.1%)	252	0	0	Yes

Legend: ^a^ I148T included in CFTR-17TnUpdate was not counted as a mutation if not in cis with c.3067-72del6 (l.n. 3199del6); c.1210(-12)T_n_ site in intron 9 (l.n.IVS8-T_n_) without data on the associated TG repeat was not counted as a mutation.

**Table 2:** Mutations found in the analyzed cohort of 738 Polish CF patients, sorted according to the position in the gene.

Exon / intron (legacy)	Exon / intron (Ensembl)	Protein change	SVM value	cDNA (HGVS nomenclature)	gDNA (cDNA +132 bp)	Number of PL CF chromosomes	Reference a	Mutations in trans
**Pathogenic mutations**								
**1**	**1**	L15Ffs10X		c.43delC	175delC	1	CFMDB	1717-1G>A
**2**	**2**	G27V	−1.92	c.80G>T	212G>T	1	Novel	F508del
**2**	**2**	S18RfsX16		c.54-5940_273 +10250del21kb	exon2,3del21kb	66	IL19	various CF mutations
**i2**	**i2**	IVS2_Donor		c.164+1G>A	296+1G>A	3	CFMDB	various CF mutations
**3**	**3**	G85E	−2.61	c.254G>A	386G>A	1	IL17	unknown
**3**	**3**	E60X		c.178G>T	310G>T	0	IL17	x
**3**	**3**	L88IfsX22		c.262_263delTT	394delTT	0	IL17	x
**4**	**4**	E92K	−1.92	c.274G>A	406G>A	2	CFMDB	c.164+1G>A; c.2051-2AA>G
**4**	**4**	L101X		c.302T>G	434T>G	1	CFMDB	c.3717+12191C>T
**4**	**4**	K114IfsX5		c.341_353del13bp	473del13bp	1	Novel	F508del
**4**	**4**	R117H	−0.35	c.350G>A	482G>A	5	IL17	F508del; 2x unknown
**4**	**4**	R117C	−2.07	c.349C>T	481C>T	2	CFMDB	S1206X;1x unknown
**4**	**4**	L137_L138insT		c.412_413insACT	L138ins	1	CFMDB	F508del
**4**	**4**	R153I	−2.61	c.458G>T	590G>T	2	Novel	F508del; c.3527delC
**i4**	**i4**	IVS4_Donor		c.489+1G>T	621+1G>T	5	IL17	F508del; c.489+1G>T
**5**	**5**	L165X		c.494T>A	626T>A	1	Novel	F508del
**i5**	**i5**	IVS5_Donor		c.579+1G>T	711+1G>T	0	IL19	x
**i5**	**i5**	IVS5_Donor		c.579+3A>G	711+3A>G	2	CFMDB	2,3del21kb; c.2052-3insA
**i5**	**i5**	IVS5_Donor		c.579+5G>A	711+5G>A	0	IL17	x
**7**	**8**	F311L	−0.90	c.933C>G	965C>G	2	CFMDB	2x F508
**7**	**8**	G314R	−0.58	c.940G>A	1072G>A	4	CFMDB	various CF mutations
**7**	**8**	F316LfsX12		c.948delT	1078delT	1	IL17	unkown
**7**	**8**	R334W	−2.41	c.1000C>T	1132C>T	6	IL17	various CF mutations
**7**	**8**	I336K	−2.07	c.1007T>A	1139T>A	2	CFMDB	2,3de21kb; F508del
**7**	**8**	R347P	−2.27	c.1040G>C	1172G>C	11	IL17	various CF mutations
**i7**	**i8**	IVS8_Donor		c.1116+2T>A	1248+2T>A	1	Novel	Q1412X
**9**	**10**	A455E	−2.61	c.1364C>A	1496C>A	0	IL17	x
**i9**	**i10**	IVS10_Donor		c.1392+1G>A	1524+1G>A	1	CFMDB	c.3816-7delGT
**10**	**11**	S466X		c.1397C>G	1529C>G	1	CFMDB	G542X
**10**	**11**	I507del		c.1519_1521delATC	1651delATC	2	IL19	F508del
**10**	**11**	F508del		c.1521_1523delCTT	1654delCTT	805	IL19	various CF mutations
**i10**	**i11**	IVS11_Acceptor		c.1585-1G>A	1717-1G>A	27	IL19	various CF mutations
**11**	**12**	G542X		c.1624G>T	1756G>T	25	IL19	various CF mutations
**11**	**12**	G551D	−1.24	c.1624G>T	1756G>T	5	IL19	various CF mutations
**11**	**12**	Q552X		c.1654C>T	1786C>T	0	IL19	x
**11**	**12**	R553X		c.1657C>T	1789C>T	14	IL19	various CF mutations
**11**	**12**	R560T	−1.92	c.1679G>C	1811G>C	0	IL19	x
**i12**	**i13**	IVS13_Donor		c.1766+1G>A	1898+1G>A	6	IL19	various CF mutations
**i12**	**i13**	IVS13_Donor		c.1766+1G>C	1898+1G>C	1	CFMDB	F508del
**13**	**14**	H620P	−1.73	c.1859A>C	1991A>C	1	CFMDB	F508del
**13**	**14**	R668C//G576A	−1.61//1.73	c.2002C>T//c.1727G>C	2134C>T//1859G>C	5 ^b^	CFMDB//rs1800098	c.1585-1G>A; 4 unknown
**13**	**14**	L671X		c.2012delT	2143delT	27	IL17	various CF mutations
**13**	**14**	K684SfsX38		c.2051_2052delAAinsG	2183AA>G	10	IL17	various CF mutations
**13**	**14**	K684NfsX38		c.2052delA	2184delA	0	IL17	x
**13**	**14**	Q685TfsX4		c.2052_2053insA	2184insA	15	CFMDB	various CF mutations^c^, 1 unknown
**13**	**14**	L732X		c.2195T>G	2327T>G	1	CFMDB	F508del
**14A**	**15**	R851X		c.2551C>T	2683C>T	3	CFMDB	various CF mutations
**14A**	**15**	I864SfsX28		c.2589_2599del11bp	2721del11bp	2	CFMDB	F508del; 2,3del21kb
**i14B**	**i16**	IVS16_Donor		c.2657+2_2657+3insA	2789+2insA	1	CFMDB	F508del
**i14B**	**i16**	IVS16_Donor		c.2657+5G>A	2789+5G>A	0	IL17	unkown
**15**	**17**	Y919C	−1.02	c.2756A>G	2888A>G	1	CFMDB	unknown
**15**	**17**	H939HfsX27		c.2817_2820delTACTC	2949delTACTC	1	Novel	unkown
**i15**	**i17**	IVS17_Donor		c.2908+3A>C	3040+3A>C	1	Novel	F508del
**i16**	**i18**	IVS18_Donor		c.2988+1G>A	3120+1G>A	0	IL19	x
**17A**	**19**	I1023_V1024del		c.3067_3072delATAGTG	3199del6	0	IL19	x
**i17A**	**i19**	IVS19		c.3140-26A>G	3272-26A>G	9	IL19	various CF mutations
**17B**	**20**	L1065R	−1.90	c.3194T>G	3326T>G	1	CFMDB	F508del
**17B**	**20**	Y1092X		c.3276C>A	3408C>A	1	CFMDB	R334W
**i18**	**i21**	IVS21_Donor		c.3468+2_3468+3insT	3600+2insT	11	CFMDB	various CF mutations^d^, 1 unknown
**18**	**21**	E1126EfsX7		c.3376_3379delGAAG	3508delGAAG	1	Novel	F508del
**19**	**22**	R1158X		c.3472C>T	3604C>T	2	CFMDB	F508del; R553X
**19**	**22**	R1162X		c.3484C>T	3616C>T	1	IL17	F508del
**19**	**22**	L1177SfsX15		c.3528delC	3659delC	4	IL17	various CF mutations
**19**	**22**	S1206X		c.3617C>A	3749C>A	1	CFMDB	R117C
**i19**	**i22**	IVS22		c.3717+12191C>T	3849+10kbC>T	58	IL17	various CF mutations
**20**	**23**	G1244R	−2.62	c.3730G>C	3862G>C	1	CFMDB	F508del
**20**	**23**	S1251N	−2.28	c.3752G>A	3884G>A	0	IL19	x
**20**	**23**	L1258FfsX7		c.3773_3774insT	3905insT	0	IL19	x
**20**	**23**	V1272VfsX28		c.3816_3817delGT	3944delGT	1	CFMDB	c.1392+1G>A
**20**	**23**	W1282X		c.3846G>A	3978G>A	9	IL19	various CF mutations
**21**	**24**	N1303K	−2.62	c.3909C>G	4041C>G	18	IL19	various CF mutations
**22**	**25**	V1327X		c.3979delG	4111delG	1	Novel	F508del
**22**	**25**	S1347PfsX13		c.4035_4038dupCCTA	c.4167dupCCTA	1	CFMDB	2,3del21kb
**23**	**26**	Q1382X		c.4144C>T	4276C>T	1	CFMDB	F508del
**23**	**26**	Q1412X		c.4234C>T	4366C>T	2	CFMDB	F508del; c.1116+2T>A
**i23**	**i26**	IVS26_Donor		c.4242+1G>T	4374+1G>T	1	CFMDB	F508del
**Sequence changes of uncertain pathogenic effect, tentatively counted as mutations**								
**6A**	**6**	E217G	0.30	c.650A>G	782A>G	1	CFMDB; rs1219109046	unknown
**7**	**8**	R352Q	−0.01	c.1055G>A	1187G>A	1	CFMDB; rs121908753	F508del
**7**	**8**	Q359R	0.33	c.1076A>G	1208A>G	1	CFMDB	F508del
**i8**	**i9**	IVS9		c.1210-12T_5__1210-34_35 (TG)_12_	1332-12T_n__-34TG_m_	6	CFMDB	F508del; 3x unknown
**i8**	**i9**	IVS9		c.1210-12T_5__1210-34_35 (TG)_13_	1332-12T_n__-34TG_m_	2	CFMDB	2143delT; 1x unknown
**i8**	**i9**	IVS9		c.1210-12T_8_	1332-12T_n_	1	Novel	unknown
**10**	**11**	I506V	−0.21	c.1516A>G	1648A>G	1	CFMDB; rs1800091	unknown
**12**	**13**	V562L	0.79	c.1684G>C	1816G>C	1	CFMDB; rs1800097	unknown
**13**	**14**	G723V	0.44	c.2168G>T	2300G>T	1	CFMDB; rs200531709	unknown
**15**	**17**	D924N	0.03	c.2770G>A	2902G>A	1	CFMDB; rs201759207	unknown
**15**	**17**	L967S	0.27	c.2900T>C	3032T>C	1	CFMDB; rs1800110	unknown
**18**	**21**	D1152H	0.50	c.3454G>C	3586G>C	1	CFMDB; rs75541969	F508del
**Sequence changes considered as lacking pathogenic effect**								
**4**	**4**	I148T	2.04	c.443T>U	575T>U	4	IL19^e^	unknown
**13**	**14**	I752V	0.35	c.2254A>G	2386A>G	1	Novel^f^	F508
**15**	**17**	S912L	2.12	c.2735C>T	2867C>T	1	CFMDB^g^ ; rs121909034	F508

Legend: ^a^ IL19 i 17 – mutations included in the INNOLiPA tests (see below); CFMDB – non-INNOLiPA mutations present in the CTFR mutation database; novel – mutations first reported in this study; ^b^ in three chromosomes R668C with G576A in trans; ^c^ F508del, c.1585-1G>A, G542X, N1303K or c.579+3A>G; ^d^ F508del, G542X, R553X or N1303K; ^e^ not pathogenic if not in cis with c.3067-72del6 (l.n.3199del6); ^f^ not pathogenic – see explanation the text; ^g^ not pathogenic if not in cis with G1244V.

a Mutations detected by two INNOLiPA_CFTR tests (legacy names): IL19 (INNOLiPA_*CFTR*19): F508del; G542X; N1303K; W1282X; G551D; 1717-1G>A; R553X; *CFTR*dele2,3(21kb); I507del; 711+1G>T; 3272-26A>G; 3905insT; R560T; 1898+1G>A; S1251N; I148T; 3199del6; 3120+1G>A; Q552X.

IL17 (INNOLiPA_*CFTR*17_TnUpdate): 621+1G>T; 3849+10kbC>T; 2183AA>G; 394delTT; 2789+5G>A; R1162X; 3659delC; R117H; R334W; R347P; G85E; 1078delT; A455E; 2143delT; E60X; 2184delA; 711+5G>A; polymorphism 5T/7T/9T.

All the CF patients without two IL mutations were analyzed further using PCR - SSCP/HD - sequencing approach. Among fifty-six non- IL mutations revealed in 99 patients, forty-five were already reported in the CFMDB, and eleven were novel, never described before (Table 2). All migration variants detected by SSCP/HD were confirmed /explained by dideoxy sequencing.

Two of the non-IL CFMDB mutations were relatively frequent. The c.2052_2053insA (legacy name, l.n.2184insA) was found in 15 (1.0%) analyzed chromosomes, and the c.3468+2_3468+3insT (l.n.3600+2insT) – in 11 (0.7%). Both mutations were in a compound heterozygosity, in most cases with F508del, and in singular patients with another CF mutation (Table 2); in two patients, no mutations in trans were found. In the Clinical and Functional Translation of CFTR database (CFTR2; www.cftr2.org), c.2052_2053insA has been reported as causing pancreatic insufficiency (PI); here, most of the patients were PI but some were pancreatic sufficient (PS); the same was observed for patients carrying c.3468+2_3468+3insT.

Two other CFMDB mutations affected the c.1210-12T_n__1210-34_35TG_m_ site in intron 9 (l.n.IVS8-T_n_/TG_m_). Specific SSCP/HD patterns (confirmed by sequencing) allowed unambiguous differentiation of the T_n_ alleles associated with variable size of the adjacent TG repeat. Among nineteen T_5_ alleles found in the whole analyzed cohort, eight (42%) were in cis with TG_12–13_; these compound alleles were not found in 300 analyzed control chromosomes. The T_5__TG_12_ combination was found in six patients (in three with F508del in trans, in three as the only mutation identified). The T_5__TG_13_ was found in two patients (in one as the only mutation, in one with c.2012delT in trans). The IVS9 T_5__TG_12–13_ compound alleles have been described in the CFTR2 database as having “varying consequences”. Here, the CF manifestation in the majority of patients carrying these alleles was relatively mild.

Of the remaining non-IL CFMDB mutations, 13 were found on 2–5 chromosomes each, and 28 were found on a single chromosome each. Eleven were amino acid substitutions whose deleterious consequence was not supported by strong negative SVM (Support Vector Machine) values. Three of them (R352Q, Q359R and D1152H) were in a compound heterozygosity with F508del, six (E217G, I506, V562L, G723V, D924N and L967S) had no accompanying mutation in trans. These nine substitutions were tentatively considered causative, but their pathogenic character requires further studies. Another substitution, G576A (SVM +1.73), was found in three patients, in cis with a deleterious R668C allele (SVM -1.61); the latter was also present without G576A, in two patients (in one with c.1585-1G>A in trans). In the UMD-CFTR database (www.umd.be/CFTR), G576A and R668C have been reported in cis; in the CFTR2 database both mutations are described as having “varying consequences”. Three of our patients carrying R668C were PI, and two appeared PS; PS/PI status was independent on the presence of G576A. We considered R668C a pathogenic mutation, and G576A – an associated element of a compound allele. Finally, S912L (SVM +2.12), found in a single chromosome with F508del in trans, has been reported in the CFMDB as pathogenic only if in cis with c.3067–3072del6; since the latter was not found in the analyzed patient, we considered S912L a neutral polymorphism.

Of the eleven novel changes, seven affected the length of the CFTR protein by: (i) introducing a premature STOP codon (two); (ii) introducing a frameshift or changing the protein length (three); (iii) changing the conserved splicing site positions (two); all were considered pathogenic mutations. They were present in a single patient each (major manifestation indicated in parentheses), and most were in trans with F508del: L165X (PI; chloride values: 88 and 90 mmol/L); V1327X (meconium ileus, MI; no chloride data available); c.341_353del13bp (PI; chloride values: 131, 143 and 147 mmol/L); c.3376_3379delGAAG (PI; chloride values: 97 and 90); c.2908+3A>C (PS; chloride values: 117, 119 and 182 mmol/L). The c.1116+2T>A (no chloride data available) was in trans with Q1412X; for the c.2817_2820delTACTC (PS; normal chloride values: 33 and 52 mmol/L), no second mutation was identified.

In case of three novel amino acid substitutions (G27V, R153I and I752V), the possibility that a change represented a non-pathological polymorphism was examined. None of these alleles was found in 300 healthy chromosomes from Polish general population, neither reported in the NCBI database for human single nucleotide polymorphisms, which encompassed data from the 1000Genome project (www.ncbi.nlm.nih.gov/SNP; build 137). The SVM values indicated strongly deleterious effect of both R153I and G27V on the protein stability (−2.61 and −1.92, respectively), and both positions were conserved in the comparative analysis with orthologues from several Eutherian species (www.ensembl.org); R153I and G27V were therefore assumed to be pathogenic mutations. R153I was found in two unrelated patients with pulmonary manifestation and a known CF mutation on another allele (F508del or c.3528delC); the first patient had elevated chloride values (94 and 98 mmol/L), and no chloride data were available for the second patient. G27V was found in a single patient with PI and pulmonary symptoms (chloride values >100 mmol/L), and was accompanied by F508del on another allele. The deleterious consequence of I752V (found in a single patient with F508del on another allele) was not supported by the SVM value (+0.35); the patient was PS and had ambiguous chloride values (45, 64 and 83 mmol/L). In addition, I752 (although present in Primates) was not conserved in other Mammalian species. I752V was therefore considered to represent a rare neutral polymorphism rather than a pathogenic mutation.

An atypical allele of the length polymorphism in intron 9, with an even number of T-s associated in cis with the TG_12_, IVS9 T_8__TG_12_, was identified in one patient with MI and failure to thrive. No mutation in trans was found and no RNA was available from the patient to experimentally confirm the effect of this mutation on *CFTR* expression. However, given that a similar change (T_6_ allele) was reported in the UMD-CFTR database as pathogenic, we tentatively considered the T_8_ allele to be a CF mutation.

MLPA (multiplex ligation-dependent probe amplification) analysis was performed to detect the potential presence of large exonic deletions in 58 patients with only one mutation and in 46 of 100 patients with no pathogenic mutations identified in the IL tests combined with the SSCP/HD screening. No changes indicative of the presence of unknown large exonic deletions were identified.

## Discussion

Using both *CFTR_19* and *CFTR_17TnUpdate* tests allowed identification of *CFTR* mutations in 75.5% of the CF chromosomes. SSCP/HD-based screening detected 53 more sequence changes assumed to be pathogenic mutations. After the extended screening of the CFTR coding sequence, the second mutation was found in 71 of the 109 patients with one IL mutation (Table 3). Among the 126 patients without IL mutations, one mutation was found in 20 and two in 6 individuals. The residual proportion of CF patients with none of the mutations detected was 13.6% (100/738), i.e. 3.5% less than after using only IL tests. The combined efficiency of *CFTR* mutations detection (82.5%) represented 7.0% increase compared to the IL tests alone. These results indicate that IL panels, which are among the most popular CF-diagnostic tests used in Europe, may not optimal for the mutation detection in PL population. The most frequent non-IL alleles identified in this study (i.e. c.2052_2053insA; c.3468+2_3468+3insT; IVS9 T_5__TG_12–13_; R668C; G314R) should be included in the PL population screening panel. Mutation distribution described herein is also relevant to the Polish; according to the Polish Press Agency, the rapport of the Polish Ministry of Foreign Affairs issued in July 2013 estimated the size of Polish diaspora at over 18 million people, with the major centers in US, Canada, Germany, UK and other countries (see also www.wikipedia.org/wiki/Polish_diaspora). The aforementioned mutations could be considered when testing these populations.

**Table 3:** Comparison of the efficiency of mutation detection efficiency using INNOLiPA tests and teh follow-up screening (SSCP/HD and sequencing).

Overall mutations detected in a patient	Proportion of patients	CF mutations per patient detected in INNOLiPA tests	Additional CF alleles detected in the follow-up phase	Number of CF patients
**both**	**78.6%**	both	n.a.	503
**“**	**“**	one	1	71^a^
**“**	**“**	none	2	6
**one**	**7.8%**	none	1	20^b,c^
**“**	**“**	one	0	38^c^
**none**	**13.6%**	none	0	38^c^+62

Legend: ^a^ including six alleles with T_5__TG_12–13_ in intron 9; ^b^ including four alleles with T_5or8__TG_12–13_ in intron 9; ^c^ also analyzed by MLPA

On the other hand, even the extended screening procedure left over 17% of *CFTR* mutations unidentified. It has to be kept in mind that, for budgetary reasons, sequencing was performed when an altered migration pattern of an amplicon was detected in the SSCP/HD analysis. Our long experience with the SSCP/HD technique indicates that using appropriate conditions allows detecting ∼85–90% of the existing sequence changes. Applying this estimate to the 158 chromosomes with no identified mutation, one may assume that 10–15% of them (∼16–24 chromosomes) could harbor sequence change undetected due to technical reasons. Nevertheless, this would still leave over 130 chromosomes with no identified mutation. The presence of large novel exonic deletions, not detectable using SSCP/HD-based approach, was excluded by MLPA analysis performed in ∼60% of the chromosomes with no identified mutation. Undetected mutations could therefore be located in the gene regions, which were not targeted in the screening procedure, such as deep intronic sequences or regulatory regions [12]. Also, when estimating mutation detection efficiency, the accuracy of the clinical diagnosis is an important issue, as discussed below.

Heterogeneity of the clinical manifestation may render clinical diagnosis of some cases difficult. This problem had already been noted in one of the previous studies on *CFTR* mutations, where patients had been divided into groups of “CF classical, atypical and doubtful” [7]. In this context it is also worth mentioning that the estimates of CF incidence in PL population differ, very likely reflecting differences in diagnostic schemes [2]. The most-cited source, indicating the incidence of 1∶2300, is now 40 years old [13]. The more recent estimates provide much lower values, ranging from 1∶5000 [14], 1∶6000 cited in WHO 2002 report [15] to 1∶7500 for Southeastern Poland estimated for a 1-year period of 2009–2010 [16]. Some of the discrepancies in the mutation detection efficiency across populations and studies may reflect this type of inaccuracies.

**Figure 1:**
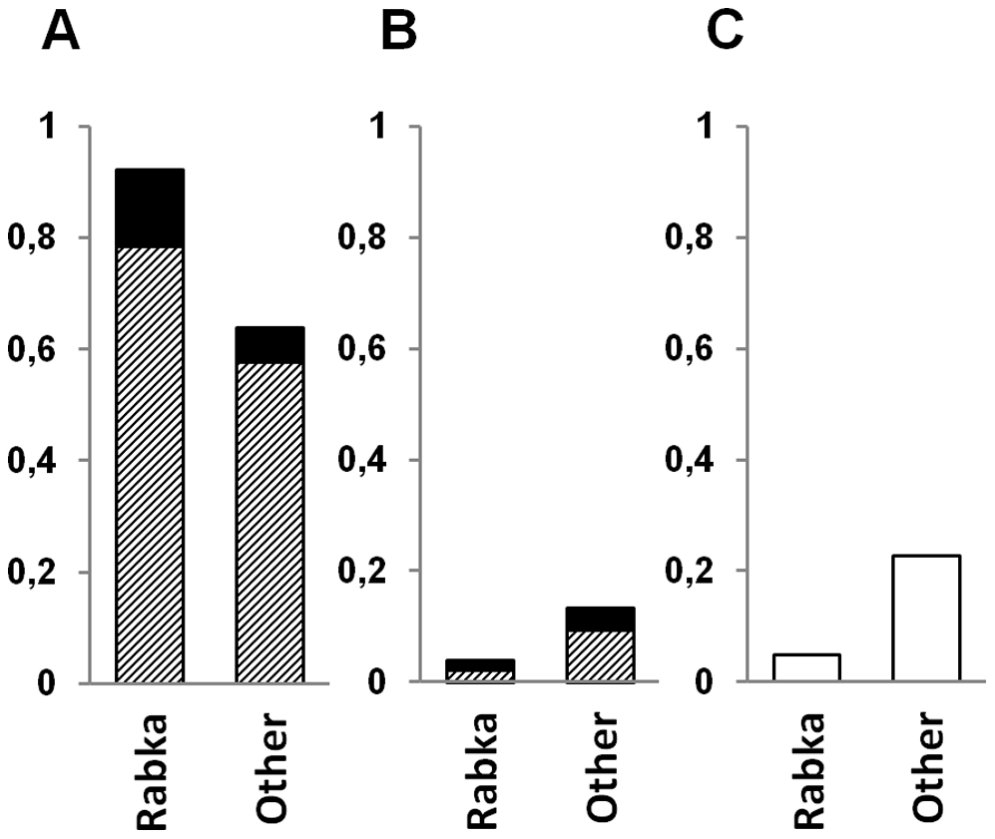
*CFTR* mutation detection efficiency in PL CF patients from different health centers. A. Both mutations identified. B. One mutation identified. C. No mutation identified. Diagonal stripes – INNOLiPA mutations; solid black – non-INNOLiPA mutations. Rabka – Institute of Tuberculosis and Lung Diseases in Rabka; Other – other health care centers in Poland.

To illustrate potential impact of the care center on the correct clinical diagnosis and thus on the efficiency of mutation detection, we compared the results obtained for two subgroups of our cohort (Fig. 1): patients from the national CF reference center (Institute of Tuberculosis and Lung Diseases in Rabka; N = 368) and from other health centers (general pediatric hospitals in Poznan and other cities, excluding Warsaw (see below); N = 370). The success of the IL tests (detection of both mutations) was 79% in Rabka and 58% in other centers (p<0.0001; Pearson’s chi square test). After the extended gene screening, the number of patients with no mutations detected remained significantly (p<0.0001) higher in patients from the general pediatric centers (∼23%) than in patients from the specialized CF center in Rabka (∼5%). The observed discrepancy can be interpreted as indicating the lower rate of a successful clinical CF diagnosis outside the reference center. Of note, among the patients from Rabka with only one or with no mutation found, ∼80% had high sweat chloride values; in the corresponding group from the peripheral centers, high chloride values were reported only in ∼50% of the patients, while the other half had ambiguous chloride values or no test results had been reported.

The efficiency of non-IL mutations detection was almost 5 times higher in the patients with one IL mutation than in those with no IL mutation (non-IL mutation found in ∼65% and ∼13% of the chromosomes, respectively, see Table 3). This discrepancy appears consistent with the scenario that some individuals of the former group (composed mostly of patients from outside of Rabka) were in fact misdiagnosed as CF. In addition, in over 20% of the patients with no mutation found, the clinical diagnosis was based on the presence of MI. While MI occurs in 10–20% of all CF patients at birth [17], a considerable proportion (20–50%) of newborns with MI may not in fact have CF [18]–[19].

In conclusion, taking into account that up to ∼10% of the whole analyzed cohort could have been incorrectly diagnosed as CF, it is possible that the real efficiency of mutation detection would be higher than the estimated 85%; similar correction would apply to the estimated mutation frequencies.

Our study group consisted mostly of CF patients from the Western and Southern PL. In order to assess the mutation spectrum in the whole country, we compared our data with those obtained in the Institute of Mother and Child in Warsaw, where most of the patients were from the Central and Northeastern Poland (Prof. Jerzy Bal, personal communication). The data (based on direct sequencing of the coding region) were only available for patients (N = 480) with both mutations detected (the total number of analyzed CF patients and of patients with only one mutation detected were not provided). To compare data from both laboratories, we calculated allele frequencies in a subset of our patients, with both mutations identified. F508del frequency in this subset was significantly higher than in the full cohort (67.2% vs 54.5%, p = 0.000; Pearsons’s chi square), which most probably results from the possible presence of misdiagnosed patients, as discussed above; the adjusted frequencies for other mutations did not differ between both subsets of our cohort. Three mutations in our reduced data set were significantly (p<0.03) less frequent than in Warsaw (F508del: 67.2% vs 71.8%; G542X: 1.98% vs 2.92%; N1303K: 1.47% vs 2.92%), while two mutations were significantly more frequent (c.3468+2_3468+3insT: 0.86% vs 0.10%; c.489+1G>T: 0.43% vs 0%; G314R: 0.34% vs 0%). The discrepancies between data from our laboratory and from Warsaw may reflect regional differences in samples origin.

The most frequent *CFTR* mutations found in PL patients (>0.1%, as suggested in the recommendations regarding selection of mutation panels [20]), listed in Table 4, were compared to those reported for several Central and Southeastern European countries [21]–[29]. The overall efficiency of the IL tests in Polish patients (75.5%; 87% in Rabka and 65% in Polish peripheral centers) was within the range reported for other populations (61–90%; see Table 4). Please note, that the highest efficiencies of testing for INNOLiPA mutations have been reported in the countries with a long-standing tradition of genetic CF testing. These data lend even further support to the observation that the reported efficiency of genetic tests in a given population strongly depends on the quality of clinical diagnostics performed in referring care centers from a given country or region.

**Table 4:** Distribution of the most frequent CFTR mutations detected in CF patients from Poland, compared with Central and Southeastern European populations.

*Mutations^a^*	*Poland*	*Czechs*	*Slovakia^c^*	*Germany*	*Lithuania*	*W. Ukraine*	*E. Hungary*	*Romania^c^*	*Bulgaria*	*Serbia*	*Greece*
*Number of chromosomes*	*1476*	*1200*	*856*	*700*	*98*	*264*	*80*	*256*	*208*	*352*	*874*
F508del	**54.54^b^**	67.42^d^	66.80^d^	72.00^d^	52.0	54.17	70.00	56.3	65.38^d^	72.28^d^	53.40
exon2,3del21kb (l.n.CFTRdele2,3_21kb)	**4.47**	5.75	2.26	1.2^f^	2.0	4.17	5.00	1.6	NA	0^e^	0.34^e^
c.3717+12191C>T (l.n.3849+10kbC>T)	**3.93**	1.67^e^	4.28	1.00^e^	NA	0.76	0	0.4^e^	1.44	0^e^	0.11^e^
c.2012delT (l.n.2143delT)	**1.83**	0.92	1.10	0.71	0	1.14	0	0^e^	0	0^e^	0^e^
c.1585-1G>A (l.n.1717-1G>A)	**1.83**	0.33^e^	NA	0.86	0	0.38	1.25	0.4	0	0^e^	0^e^
G542X	**1.69**	2.00	4.06^d^	1.43	0	2.65	3.75	3.9	3.37	2.57	3.90^d^
R347P	**1.57**	0.92	1.10	1.57	0	0	1.25	NA	1.44	0 ^e^	0.11^e^
N1303K	**1.22**	2.42	2.03	2.29	2.0	4.92^d^	5.00	0.8	6.73^d^	0	2.63
**c.2052-2053insA (l.n.2184insA)**	**1.02**	0.42	1.58	0.57	0	7.20^d^	5.00^d^	0	0.48	0.28	0^e^
R553X	**0.95**	0.50	0.90	2.29	4.2^d^	0.38	0	NA	0	0	0
**c.3468+2−3insT (l.n.3600+2insT)**	**0.75**	0.25	NA	0^e^	0	NA	0	NA	0	0	0^e^
c.2051–2052AA>G (l.n.2183AA>G)	**0.68**	0.08	NA	0.57	0	0.38	0	0.8	0	0	1.38
W1282X	**0.61**	0.58	0.50	0.71	1.0	2.27	0	2.3^d^	0.96	0	0.67
c.3140-26A>G (l.n.3272-26A>G)	**0.61**	0.67	0.50	0.86	0	0.76	0	0.4	0	0	0.81
**l.n.IVS8 T_5__TG_12–13_**	**0.54**	NA	NA	NA	0	NA	NA	NA	NA	0	NA
R334W	**0.41**	0.25	NA	0.29	0	0.76	0	0.4	0	0.28	0.81
c.1766+1G>A (l.n.1898+1G>A)	**0.41**	1.42^d^	0.50	0	0	1.14	0	NA	0	0	0.11
c.489+1G>T (l.n.621+1G>T)	**0.34**	0.42	NA	0.14	0	0.76	0	0.8	0	2.86^d^	5.72^d^
R117H	**0.34**	NA	NA	0.29	0	0	0	0.4	0	0	0.23
G551D	**0.34**	2.91^d^	0.50	1.00	0	0	0	0	0	0	0.34
**G314R**	**0.37**	0	NA	0	0	0	0	NA	0	0	0
**R668C**	**0.34**	0	NA	0	0	0	0	NA	0	0	0
c.3528delC (l.n.3659delC)	**0.27**	0.17	NA	0.57	0	0	0	NA	0	0	0
**c.164+1G>A (l.n.296+1G>A)**	**0.20**	0.08	NA	0	0	0	0	NA	0	0	0
R851X	**0.20**	0.08	NA	0	0	0	0	NA	0	0	0
**I336K**	**0.14**	0.58	NA	0.45	0	0	0	NA	0	0	0
**R1158X**	**0.14**	0.08	NA	0	0	0	0	NA	0	0	1.03
**E92K**	**0.14**	0.08	NA	0	0	0.38	0	NA	0	0	0
**R153I**	**0.14**	0	NA	0	0	0	0	NA	0	0	0
**c.579+3A>G (l.n.711+3A>G)**	**0.14**	0.17	NA	0	0	0	0	NA	0	0	0.69
**c.2589–2599del11bp (l.n.2721-31del11bp)**	**0.14**	0.08	NA	0	0	0.38	0	NA	0	0	0
I507del	**0.14**	0.08	NA	0.15	0	0	0	0	0	0.28	0.69
**R117C**	**0.14**	0.08	NA	0.15	0	0	0	NA	0	0	0.23
**F311L**	**0.14**	0	NA	0	0	0	0	NA	0	0	0
**Q1412X**	**0.14**	0	NA	0	0	0	0	NA	0	0	0
Other reported	**1.52**	8.51	NA	7.10	2.0	1.14	7.50	3.8	12,03	4.28	17.83
Not detected	**17.5**	0.50	13.89	4.57	35.8	16.29	6.25	27.7	8.17	17.43	9.15
**Estimated efficiency of INNOLiPA tests**	**75.5**	**89.9**	**84.0**	**88.7**	**61.2**	**74.6**	**87.5**	**69.1**	**80.3**	**78.7**	**73.3**

Legend: Data are given in %. ^a^ non-INNOLiPA mutations are in bold, and a novel mutation is underlined; ^b^ very close to the earlier estimate of 54% based on a much smaller study group of PL patients [7]; ^c^ only selected segments of the gene have been screened; ^d^ frequency significantly higher or ^e^ lower than in Polish cohort (p<0.005, Pearsons’s chi square); ^f^ [5]; NA– not analyzed/not available; Poland (mostly Southern and Western Poland; this study); Czech Republic [21]; Slovakia (based on [21]); Germany [22]; Lithuania [23]; Western Ukraine [24]; East Hungary [25]; Romania [26]; Bulgaria [27]; Serbia [28]; Greece [29].

The frequency of F508del (∼54.5%), was similar to the estimates for Western Ukrainians, Lithuanians, Romanians and Greeks but significantly (p<0.006; Pearsons’s chi square) lower than for Germans, Czechs, Slovaks, Bulgarians, and Serbs; this shows that the decreasing North-South gradient of F508del frequency across Europe [4] does not hold in Eastern Slav populations. The relatively high frequency of exon2.3del21kb alias *CFTR*dele2,3(21 kb) (∼4.5%) was consistent with its Slavic origin [30]. The frequency of Israeli c.3717+12191C>T (l.n.3849+10kbC>T) [4] was significantly elevated in Poland (3.9%) and Slovakia compared to most of the examined populations (Czechs, Germany, Romania, Serbia, Greece, p<0.005), possibly indicating the Ashkenazi-Jewish contribution; in contrast, the frequency of another Israeli mutation, W1282X [4], [12], was significantly lower in Poland (0.61%) than in Romania (p<0.006). The Eastern European c.2012delT (l.n.2143delT) [12] was found in Poles at the relatively high frequency (1.83%) compared to Czechs, Slovaks, Western Ukrainians and Germans, and was absent elsewhere, including Romania, Serbia and Bulgaria; its increased frequency may suggest PL origin of the c.2012delT. The frequency of Mediterranean c.1585-1G>A (l.n.1717-1G>A) [4]–[5] was 1.83%, significantly (p<0.005) higher than in several other Slavic populations, while that of another Mediterranean mutation, N1303K [4], was 1.22%, significantly (p<0.005) lower than in Western Ukraine, Bulgaria, Serbia and Hungary.

Among non-IL mutations, the frequency of c.2052–2053insA (l.n.2184insA, ∼1.0%) was significantly (p<0.005) lower than 7.2% or 5.0% reported for Western Ukraine or Eastern Hungary, respectively [24], [25]. This confirms the proposed “Galician” origin of this mutation and indicates that Poland is at the decreasing side of its distribution. The c.3468+2_3468+3insT (originally reported in a single PL individual [7]), accounted for ∼0.75% PL CF chromosomes; so far, it has been reported only in Poles and in Czechs [21]. The regional origin of Polish patients carrying this insertion (Southeastern part of PL) and the homogeneous SNP-based background haplotype (not shown) indicate that this is probably a founder mutation. G314R, a novel missense change found in 0.37% of the studied CF chromosomes, might be another PL founder mutation. These observations would have to be confirmed by screening for c.3468+2_3468+3insT and G314R in a larger numbers of CF patients from other neighboring European populations.

The relatively high frequency (0.54%) of IVS9 T_5__TG_12–13_ (without R117H in cis) in PL patients could not be compared with other populations. While TG_12–13_ in cis with T_5_ are known to contribute to less efficient splicing of exon 10 and to abnormal phenotype [31]–[32], most of the CF tests do not determine the length of the TG repeat, and reporting the T_5_ in intron 9 (l.n.IVS8-T_5_) as a pathogenic mutation has been recommended only if in cis with R117H [12].

## Materials and Methods

### Ethics Statement

The research protocol was approved by the Ethics Committee of the Medical University in Poznan. Genetic data referred to in the manuscript were collected over many years as a component of a routine hospital diagnostic procedure. As such, they are covered by an implied consent, which – according to the local Institutional Review Board – does not require signing additional informed consent form and is documented by the fact of a patient subjecting to the diagnostic procedure. All further analyses were based on the archival data that were stored in the database with no current connection to the patients’ identifiers.

### Patients

The samples, referred for molecular diagnosis during the period of 1995–2011, originated from different care centers (predominantly Institute of Tuberculosis and Lung Diseases in Rabka, ∼50%; and pediatric clinical hospitals from Poznan, ∼25%); patients represented mostly Western and Southern PL. The initial diagnosis of CF was based on standard criteria [6], [33], which included one or more of the characteristic clinical features (most often: chronic obstruction/ infection of the respiratory tract; gastrointestinal abnormalities; failure to thrive; meconium ileus, MI; exocrine pancreatic insufficiency) and/or at least two positive sweat chloride tests in pilocarpine iontophoresis (>60 mmol/L). Chloride measurements were not collected for newborns and very young children, consistent with the well-known observation that no sweat tests are reliable during the first years of age (e.g. [34]); in particular, for most of the patients with MI no chloride data were available.

### Mutation screening

The group of unrelated CF patients (N = 738) had been sequentially examined using IL tests: *CFTR_19* and – if two mutations were not detected – *CFTR*_*17TnUpdate*. IL reverse dot blot kits rely on the DNA amplification and hybridization with a membrane-immobilized set of probes (Line Probe Assay). The probes correspond to the normal and mutated alleles, covering 36 most frequent European *CFTR* mutations. The procedure was performed as indicated by the manufacturer (InnoGenetics, Ghent, Belgium; see also [35]).

To comprehensively determine the population frequency profile of *CFTR* mutations, a follow-up molecular screening was performed. All the patients with only one IL mutation (N = 109) and with no mutation found (N = 126) were analyzed using SSCP/HD (single strand conformational polymorphism and heteroduplex) technique as described in [11]. Briefly, specific primer pairs were designed for each of the 27 *CFTR* exons, and the 5’ and 3’ UTR regions; to achieve the length of each amplicon <340 bp some exons were analyzed as overlapping fragments. For the SSCP/HD analysis, PCR-amplified segments were denatured and separated in 7 or 8% polyacrylamide (29∶1) in 0.5x or 1xTBE gels (optionally with ∼2 M urea and 10% glycerol) were run at 8–10 W for 20–40 h at RT or 4°C. Primer sequences, PCR conditions and detailed conditions used to separate each of the analyzed fragments are available from the authors upon request.

Nucleotide changes underlying all the detected SSCP/HD migration variants were resolved by direct sequencing of the PCR products (BigDyeTerminator v3.1 on an ABI Prism 3130XL Analyzer, Applied Biosystems); trace files were checked and edited using FinchTV1.3.1. (Geospiza Inc.). Sequences were evaluated manually using Chromas 1.45 software and FASTA sequence comparison algorithm (http://fasta.bioch.virginia.edu/fasta_www2).

Finally, 2/3 of the samples, in which one or both mutations remained unidentified, were examined for possible intragenic rearrangements using MLPA technique with SALSA MLPA Kit P091-B1 CFTR (MRC-Holland). This P091-B1 CFTR probemix contains probes for each of the 24 *CFTR* exons and a second probe for exons 6, 14, 17 and 24. In addition, the probemix P091-B1 contains a mutation specific that detects the wildtype allele of the F508del mutation.

The effect of amino acid changes on the protein stability was examined using SNPs3D online software (www.snps3d.org); the negative SVM value (<−0.5) was assumed to indicate a deleterious effect.

### Nomenclature of mutations

The currently recommended numbering of the gene exons (www.ensembl.org) and the protein or cDNA-based HGVS nomenclature of mutations were used throughout the text; the commonly used legacy name (l.n.), if mentioned, was given in parentheses.
