# Correction: *CFTR* Mutations Spectrum and the Efficiency of Molecular Diagnostics in Polish Cystic Fibrosis Patients

**DOI:** 10.1371/journal.pone.0105738

**Published:** 2014-08-07

**Authors:** The PLOS ONE Staff

There is an error in reference 21. The correct reference is: Křenková P, Piskáčková T, Holubová A, Balaščaková M, Krulišová V, et al. (2012) Distribution of CFTR mutations in the Czech population: positive impact of integrated clinical and laboratory expertise, detection of novel/de novo alleles and relevance for related/derived populations. *J Cyst Fibros* 2013 Sep;12(5):532-7. doi: 10.1016/j.jcf.2012.12.002
